# Meta-Analysis of Gastrointestinal Adverse Events from Tyrosine Kinase Inhibitors for Chronic Myeloid Leukemia

**DOI:** 10.3390/cancers13071643

**Published:** 2021-04-01

**Authors:** Prahathishree Mohanavelu, Mira Mutnick, Nidhi Mehra, Brandon White, Sparsh Kudrimoti, Kaci Hernandez Kluesner, Xinyu Chen, Tim Nguyen, Elaina Horlander, Helena Thenot, Vamsi Kota, Cassie S. Mitchell

**Affiliations:** 1 Laboratory for Pathology Dynamics, Department of Biomedical Engineering, Georgia Institute of Technology and Emory University School of Medicine, Atlanta, GA 30332, USA; pmohanavelu@gatech.edu (P.M.); miramutnick@gatech.edu (M.M.); nmehra3@gatech.edu (N.M.); brandonleewhitejr@gatech.edu (B.W.); skudrimoti6@gatech.edu (S.K.); kkluesner3@gatech.edu (K.H.K.); xinyuchen@gatech.edu (X.C.); tnguyen475@gatech.edu (T.N.); ehorlander3@gatech.edu (E.H.); hthenot3@gatech.edu (H.T.); 2 Hematology and Oncology, Georgia Cancer Center, Augusta University, Augusta, GA 30912, USA; vkota@augusta.edu

**Keywords:** chronic myeloid leukemia, tyrosine kinase inhibitor, gastrointestinal adverse event, quality of life

## Abstract

**Simple Summary:**

Philadelphia chromosome positive (Ph+) chronic myeloid leukemia (CML) patients treated with tyrosine kinase inhibitors (TKIs) often suffer from adverse events that negatively impact quality of life and patient therapy compliance. The purpose of this meta-analysis was to assess and compare the incidence of gastrointestinal adverse events (GI AEs), particularly in second-generation TKIs, in a very large, heterogeneous CML population. Results illustrate significant differences in GI AE profiles between different TKIs but minimal differences in patient survival. TKI AE profile should be a primary consideration when choosing an optimal, personalized TKI therapy for chronic phase CML patients without resistant mutations.

**Abstract:**

Tyrosine kinase inhibitors (TKIs) are the frontline therapy for BCR-ABL (Ph+) chronic myeloid leukemia (CML). A systematic meta-analysis of 43 peer-reviewed studies with 10,769 CML patients compared the incidence of gastrointestinal adverse events (GI AEs) in a large heterogeneous CML population as a function of TKI type. Incidence and severity of nausea, vomiting, and diarrhea were assessed for imatinib, dasatinib, bosutinib, and nilotinib. Examination of combined TKI average GI AE incidence found diarrhea most prevalent (22.5%), followed by nausea (20.6%), and vomiting (12.9%). Other TKI GI AEs included constipation (9.2%), abdominal pain (7.6%), gastrointestinal hemorrhage (3.5%), and pancreatitis (2.2%). Mean GI AE incidence was significantly different between TKIs (*p* < 0.001): bosutinib (52.9%), imatinib (24.2%), dasatinib (20.4%), and nilotinib (9.1%). Diarrhea was the most prevalent GI AE with bosutinib (79.2%) and dasatinib (28.1%), whereas nausea was most prevalent with imatinib (33.0%) and nilotinib (13.2%). Incidence of grade 3 or 4 severe GI AEs was ≤3% except severe diarrhea with bosutinib (9.5%). Unsupervised clustering revealed treatment efficacy measured by the complete cytogenetic response, major molecular response, and overall survival is driven most by disease severity, not TKI type. For patients with chronic phase CML without resistance, optimal TKI selection should consider TKI AE profile, comorbidities, and lifestyle.

## 1. Introduction

Chronic myeloid leukemia (CML) is a cancer that targets early myeloid cells in the bone marrow. It results in a building of impartially mature cells that crowd out the normal myeloid cells. The presence of a chromosomal abnormality known as the Philadelphia chromosome (Ph+), characterizes patients diagnosed with CML [1]. Ph+ results from the fusion of the Abelson (Abl) tyrosine kinase gene at chromosome 9 and the breakpoint cluster (Bcr) gene at chromosome 22. Tyrosine kinase inhibitors (TKIs) are currently the frontline treatment option for CML patients. TKIs have been proven effective in treating CML, typically defined by the achievement of a complete cytogenetic response (CCyR) where the Ph+ chromosome is no longer detectable in the bone marrow, a major molecular response (MMR) where blood levels of BCR ABL transcript are less than 1/1000th of that expected for a non-treated CML patient, as well as excellent long term overall survival (OS) >85% [2]. TKIs have not only been powerful therapies for CML, but have also been used in similar mutations found in some acute lymphocytic leukemia (ALL), acute myeloid leukemia (AML), and non-hematological cancer types, including lung, renal, and pancreatic cancers [3,4,5,6].

However, important questions about TKI usage remain unanswered. CML patient TKI treatment duration is presently recommended indefinitely or “for life” because, even with deep molecular remissions, recurrence after TKI cessation is substantial [2,7]. With such chronic use, long-term adverse events can negatively affect patient quality of life, resulting in decreases in patient therapy adherence [8,9,10] that can lead to relapse or specific drug resistance [11,12]. Characterization of TKI adverse events in heterogeneous CML populations is an important step towards optimal, personalized TKI therapy selection [9,12]. Since resistance and intolerance to imatinib, a first-generation TKI was identified, second-generation TKIs have been increasingly used in patients as both a frontline therapy and second-line therapy. Thus, more analysis of second-generation TKIs is particularly necessary as their usage has become more widespread.

CML TKI adverse event analysis is typically reported using primarily homogenous clinical trials for drug approval, where patient populations are more carefully selected, tending to be younger and healthier, or minimally with less antecedent disease or comorbidities [13]. However, typical CML populations are much more heterogeneous. These heterogeneous populations may not only have different comorbidities, but may also exhibit varying disease stages or progression of CML, including chronic phase, accelerated phase, blast crisis, resistant mutations, or previous intolerance to one or more previous TKIs. In fact, it is estimated that >25% of CML patients in their lifetime will change TKIs due to drug intolerance or resistance [14]. Large-scale data analysis of heterogeneous populations can provide helpful insights for better understanding drug adverse event profiles and considering them in the context of an individual patient and the patient’s likely response to a specific TKI. Now that multiple specific TKI drugs have shown exceptional ability to achieve a high %CCyR and %OS, treatment recommendations can shift towards the personalized selection of TKIs [15,16] to minimize adverse events, improve quality of life, and improve adherence.

Prior text mining analysis of over 24,000 CML peer-reviewed abstracts and clinical trials revealed that gastrointestinal (GI) adverse events are the most common TKI-related adverse events reported in the peer-reviewed literature text abstracts [17]. Moreover, gastrointestinal disorders are the third most common comorbidity associated with CML [18]. Therefore, GI AEs could have an important impact on optimal and personalized TKI therapy selection meant to maximize patient adherence and long-term TKI therapeutic success.

The goal of the present work was to perform large-scale aggregate analysis of heterogeneous CML cohort data to quantify, compare, and contrast the incidence of GI AEs as a function of specific TKI drug types. Specifically, how do second-generation TKIs fare, including patients who were either intolerant or resistant to the first-generation TKI, imatinib? A large systematic meta-analysis (*N* = 10,789 patients) using data from 43 published studies was conducted to quantitatively compare the incidence of GI adverse events in studies that examined a second-generation TKI alone or with an imatinib treatment arm for comparison. The large heterogeneous population intentionally includes a diverse set of CML patients in varying disease stages and differences in TKI resistance or tolerance. Through standard statistical methods and unsupervised machine learning methods, the incidence of three primary GI AEs was assessed, including diarrhea, nausea, and vomiting, along with the CML TKI treatment efficacy outcomes like %CCyR, %MMR, and %OS over a period of 12+ months.

## 2. Materials and Methods

A systematic meta-analysis was conducted using compiled data from a variety of peer-reviewed articles retrieved from PubMed and from studies available at ClinicalTrials.gov to ensure a heterogeneous patient population. The primary measured outcome of the meta-analysis was the incidence and severity of gastrointestinal adverse events (GI AEs), namely nausea, diarrhea, and vomiting, in patients taking imatinib, nilotinib, dasatinib, or bosutinib. The study intentionally comprises a diverse, heterogeneous CML patient population with patients at varying disease stages (chronic phase, accelerated phase, or blast crisis); newly or previously diagnosed CML; patients naïve to TKIs; and patients who had been previously intolerant to one or more TKIs before switching to a different TKI. A diverse patient population was utilized to ensure a realistic view of the adverse event profile for TKIs in aggregate, as well as for specific TKI drug types.

### 2.1. Text Mining to Assess Adverse Event Study Sample Size

To evaluate the breadth of data published regarding adverse events associated with the use of TKIs, searches were conducted in pubmed.gov and clinicaltrials.gov databases using the search terms ‘chronic myeloid leukemia’, ‘tyrosine kinase inhibitor’, ‘bosutinib’, ‘nilotinib’, ‘dasatinib’, or ‘imatinib’. Approximately 24,000 abstracts were retrieved and converted to a Python dictionary to perform text mining derived clustering of adverse events (see Figure S1) [17]. Each corresponding TKI cluster contained GI AEs and indicated the study sample size was sufficient for a meta-analysis [17].

### 2.2. Meta-Analysis Inclusion Criteria

The statistical meta-analysis inclusion criteria were as follows: study written in English and available in full-text; study appeared in PubMed.gov or clinicaltrials.gov using search criteria of ‘chronic myeloid leukemia’, and ‘second-generation tyrosine kinase inhibitor’ or ‘bosutinib’, ‘nilotinib’, or ‘dasatinib’; the study had at least one treatment arm with a second-generation TKI (bosutinib, nilotinib, or dasatinib); study measured at least one GI AE (nausea, vomiting, or diarrhea). Note that direct searches for imatinib were not included. Because secondary TKIs were the primary subject of the present meta-analysis, imatinib cohorts were only included from second-generation TKI studies that had an imatinib treatment arm for comparison. Constraining the inclusion criteria in this manner provided a more congruent temporal timeline of studies and corresponding clinical metrics, as well as the desired heterogeneous patient population.

Figure S2, a PRISMA diagram, illustrates a detailed breakdown of the inclusion and exclusion of articles for the meta-analysis. Notably, all included journal articles were published after 2006, given each study was required to include a second-generation TKI treatment arm. Upon exclusion of studies not meeting the stated inclusion criteria and the removal of repetitive patient cohorts (defined as studies that secondarily analyzed previously published or identical patient data), a total of 43 studies were included for data curation and analysis.

### 2.3. Data Curation

After applying inclusion criteria, GI AE data was extracted from 43 studies into a relational database using a published biocuration and quality control procedure [19] subsequently adapted to CML data recapture [20], insuring >99.8% data recapture accuracy. Records extracted included the following: clinical study information [trial identification number, total patients involved, treatments administered, and number of treatment arms], patient group information [group description, group size, median age, sex, treatment, dosage, dosage frequency, ethnicity, percentage in major molecular response (%MMR), percentage in complete cytogenetic response (%CCyR), and percentage overall survival (%OS)], and safety analysis [GI adverse event, number affected, total number of patients, percentage affected, severity, and time of measurement].

GI adverse event data were categorized by severity and treatment. The classification of a GI AE was determined by the MedDRA SOC categorization as listed in the Common Terminology Criteria for Adverse Events. The severity of GI AEs was separated into all grades during curation. Ultimately, “severe” was defined in the meta-analysis to include grades 3 and 4. The classification of the level of severity was indicated by the Common Terminology Criteria for Adverse Events protocol, published by the National Cancer Institute. For an adverse event to be considered grade 3, the event had to be “severe or medically significant but not immediately life-threatening” while grade 4 was considered “severe and life-threatening”. The population size for each adverse event varies due to the availability of reported data from the previously published studies.

### 2.4. Statistical Analysis

The primary statistical analysis determined which TKIs resulted in significantly more or less GI AEs based on AE type. A 2-sample z-test for population proportion was used to assess significant pairwise differences in the incidence of each adverse event. To account for multiple comparisons and prevent false-positive findings, the *p*-value threshold for significance was reduced from *p* < 0.01 (corresponding to an alpha of 0.01) to * *p* < 0.00160 by applying a Bonferroni correction [21].

A subset of studies with both a first-generation TKI (imatinib) treatment arm and at least one other second-generation TKI treatment arm (bosutinib, dasatinib, or nilotinib) were analyzed using the standard odds ratio method with a 95% confidence interval. The odds ratio assessed the odds of specific GI AEs (nausea, vomiting, or diarrhea) of each second-generation TKI (bosutinib, dasatinib, nilotinib) compared to imatinib. An odds ratio of 1 represented a GI AE risk equivalent to imatinib, an odds ratio greater than one represented a GI AE risk greater than imatinib, and an odds ratio less than one represented a GI AE risk less than imatinib.

Finally, unsupervised clustering methods in Python using SciKitLearn were used to further investigate potential associations based on functional treatment efficacy measures (%CCyR, %MMR, %OS) to ensure there were no drastic differences in efficacy based on TKI type amidst the very heterogeneous patient population. K-means clustering was used to segregate the data into optimal clusters via visual examination of the scree plot using the elbow method [22,23,24]. To account for outliers, density-based spatial clustering of applications with noise (DBSCAN) clustering algorithm was used to confirm the results of the k-means clustering [22,23,24,25,26].

## 3. Results

Incidence and severity of gastrointestinal adverse events (GI AEs), namely nausea, diarrhea, and vomiting, were measured and statistically analyzed in patients taking imatinib, nilotinib, dasatinib, or bosutinib. A total of 10,789 patients from 43 peer-reviewed studies examining at least one second-generation TKI with or without a comparative first-generation treatment arm (imatinib) were included in the meta-analysis. The heterogeneous meta-analysis population intentionally included patients in all disease stages (chronic, accelerated, blast crisis), as well as patients that either had resistant mutations or had previously been intolerant to or insufficiently responsive to one or more TKI. Mean or median patient ages for all included studies ranged from 36–74 years (see Table S1). For the sake of GI AE analysis, patients were not separated by CML disease stage (chronic, accelerated, or blast crisis), although, notably, patients in this analysis had previously failed imatinib or another TKI. Table 1 illustrates the incidence of GI AEs, including the number of patients experiencing all grades of GI AEs and specifically severe grades of GI AEs. Because not every study measured every GI AE, the total number of patients examined for each type of GI AE varied as shown in the denominators of the fractions in Table 1.

### 3.1. Incidence of Gastrointestinal Adverse Events

**Figure 1:**
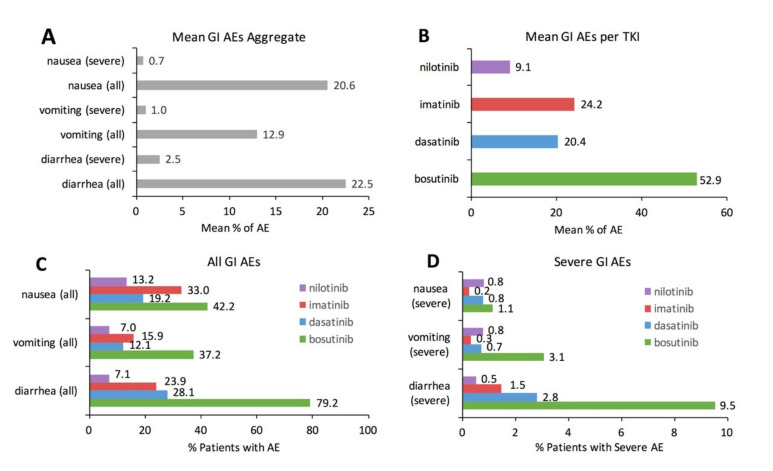
Descriptive statistics for the incidence of gastrointestinal adverse events (GI AEs) with tyrosine kinase inhibitor (TKI) therapy in chronic myeloid leukemia (CML) patients. (**A**). Mean incidence of nausea, vomiting, diarrhea aggregated across all included TKIs (bosutinib + dasatinib + imatinib + nilotinib). (**B**). Mean incidence of GI AEs for each separately included TKI (bosutinib, dasatinib, imatinib, nilotinib). (**C**). Actual or raw incidence of specific GI AEs (nausea, vomiting, and diarrhea) for each separately included TKI. (**D**). Actual or raw incidence of specific severe GI AEs (nausea, vomiting, diarrhea) for each separately included TKI. “Severe” is defined as a grade 3 or grade 4 AE based on the MedDRA SOC categorization as listed in the Common Terminology Criteria for Adverse Events.

Figure 1 visually illustrates basic descriptive statistics for the meta-analysis. Aggregate data examining the mean incidence of GI AEs for all TKIs combined illustrates that diarrhea is the most common GI AE (22.5%), followed by nausea (20.6%) and vomiting (12.9%) (Figure 1A). Aggregate data examining the mean incidence of all GI AEs combined illustrates that bosutinib had the largest mean incidence of GI AEs (52.9%), followed by imatinib (24.2%), dasatinib (20.4%), and nilotinib (9.1%) (Figure 1B). Examining specific types of GI AEs for each individual TKI illustrates differences in specific GI AE incidence as a function of individual TKI (Figure 1C). Diarrhea was the most prevalent GI AE with bosutinib (79.2%) and dasatinib (28.1%). Nausea was the most prevalent GI AE with imatinib (33.0%) and nilotinib (13.2%). The incidence of severe GI AEs, defined as grade 3 or grade 4 in severity, ranges between 0.2% to 3.1% with the exception of severe diarrhea with bosutinib (9.5%) (Figure 1D).

### 3.2. Pairwise Comparison of Tyrosine Kinase Inhibitors

**Figure 2:**
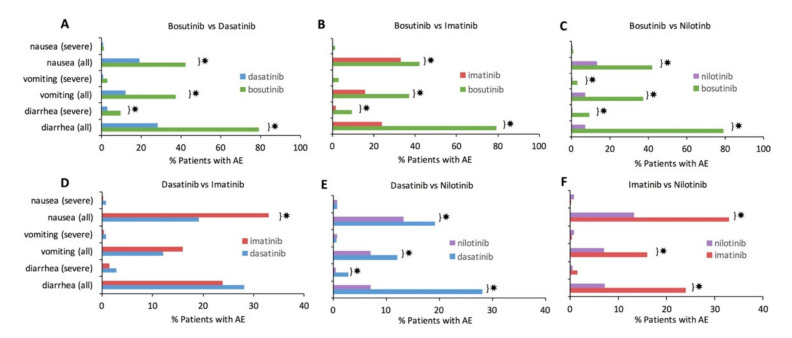
Pairwise statistical comparison of gastrointestinal adverse events (GI AEs) for each included tyrosine kinase inhibitor (TKI), including bosutinib, dasatinib, imatinib, and nilotinib. Each TKI is compared to every other TKI using z-test for the population proportion. *p*-value threshold for significance was adjusted for multiple comparisons using the Bonferroni correction, resulting in a significant *p*-value being <0.00016, which is indicated by ✸. (**A**). Bosutinib compared to dasatinib. (**B**). Bosutinib compared to imatinib. (**C**). Bosutinib compared to nilotinib. (**D**). Dasatinib compared to imatinib. (**E**). Dasatinib compared to nilotinib. (**F**). Imatinib compared to nilotinib.

Each TKI was statistically compared to every other TKI to assess significant differences in GI AE incidence. Statistically significant differences with Bonferroni correction (*p* < 0.0016) are denoted by ✸ in Figure 2. Bosutinib consistently had a significantly higher incidence of every GI AE compared to each of the other TKIs (Figure 2A–C). Nilotinib had a consistently lower incidence for most individual GI AE types compared to the other TKIs (Figure 2C,E,F). Of all the pairwise TKI comparisons, imatinib and dasatinib were most comparable in their GI AE incidence (Figure 2D); nonetheless, imatinib still had significantly more nausea than dasatinib.

### 3.3. Risk of Adverse Event Compared to Imatinib

**Figure 3:**
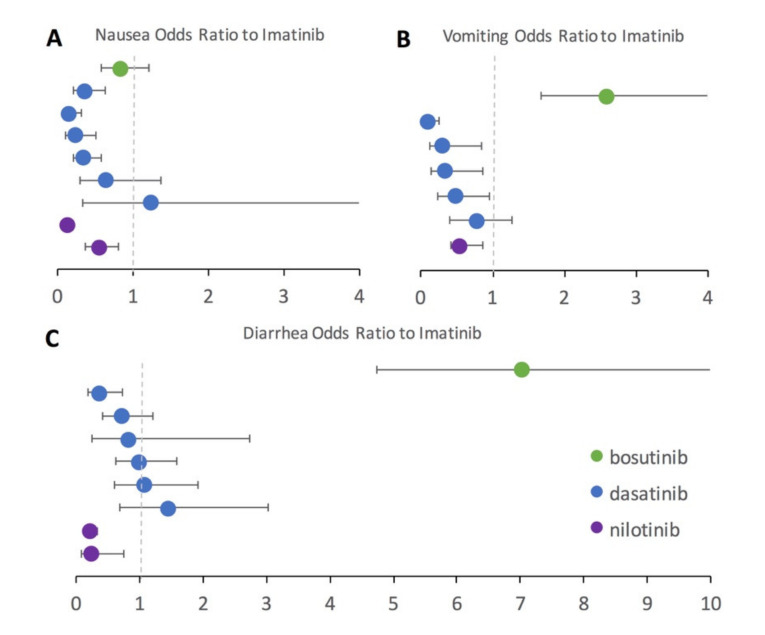
Odds ratio of gastrointestinal adverse events (GI AEs) of second-generation tyrosine kinase inhibitors (TKIs) compared to the first-generation TKI, imatinib. Shown data is from a subset of studies that included both an imatinib treatment arm and a second-generation TKI treatment arm. Error bars represent 95% confidence interval. Odds ratio equal to one is equivalent to GI AE of imatinib, odds ratio greater than 1 represents increased odds of GI AE compared to imatinib, and odds ratio less than one represents decreased odds of GI AE compared to imatinib. The vertical dotted grey line in each panel illustrates the odds ratio threshold of 1 for ease of visualization. (**A**). Nausea odds ratio to imatinib. (**B**). Vomiting odds ratio to imatinib. (**C**). Diarrhea odds ratio to imatinib.

To further assess second-generation TKIs in direct comparison to imatinib, a subset of studies that included both an imatinib treatment arm and a second-generation TKI treatment arm (bosutinib, dasatinib, or nilotinib) were examined. Using imatinib as the baseline, the odds ratio for each GI AE was computed along with the 95% confidence interval (Figure 3). Many patients taking the second generation TKIs had been previously intolerant or resistant to imatinib in the included studies. Results seen in the pairwise statistical analysis with the full dataset are generally confirmed in the odds ratio analysis performed on the subset of studies reporting results for both imatinib and a second-generation TKI treatment. Again, imatinib has more nausea than all included second-generation TKIs, which is denoted by the odds ratios falling below one (Figure 3A) for bosutinib, nilotinib, and all but a single dasatinib study. Dasatinib performed similarly to imatinib for the odds ratio of diarrhea (Figure 3C), but had less vomiting (Figure 3B) and nausea (Figure 3A). Nilotinib had lower odds than imatinib for every category of GI AE (Figure 3A–C). Bosutinib had a substantially higher odds of vomiting (Figure 3B) and especially diarrhea (Figure 3C) compared to imatinib.

### 3.4. Other Gastrointestinal Adverse Events

In the included meta-analysis studies, other GI AEs besides diarrhea, vomiting, and nausea had an aggregate incidence of 11.9%. Among the fraction of included studies in the meta-analysis that reported additional GI AEs, the mean incidence was 9.2% for constipation, 7.6% for abdominal pain, 3.5% for gastrointestinal hemorrhage, and 2.2% for pancreatitis. However, there were not enough aggregate data to statistically compare these additional GI AEs as a function of specific TKI treatment type.

### 3.5. Functional Assessment of Tyrosine Kinase Inhibitors

CML patient TKI treatment success is primarily measured by the complete cytogenetic response (CCyR), major molecular response (MMR), and overall survival (OS). A CCyR is defined as having no quantifiable cells with the BCR-ABL1 mutation in the patient’s bone marrow biopsy. A major molecular response (MMR) means that the amount of BCR-ABL gene in the blood is 1/1000th or less of what is expected in a patient with untreated CML. OS measures the percentage of patients living over the course of the study, which is typically reported as an observation or follow-up period of 12 months or more in the present studies. Assessment of functional response is important for considering TKI selection.

**Figure 4:**
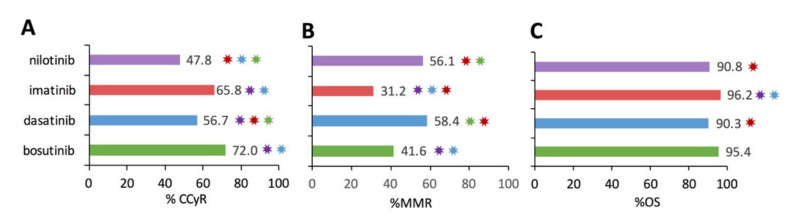
Tyrosine kinase inhibitor (TKI) treatment aggregate efficacy based on the metrics of the percentage of patients with complete cytogenetic response (%CCyR), percentage of patients with a major molecular response (%MMR), and percentage of patients overall survived (%OS) over 12+ months. Notably, imatinib patients often comprised a greater percentage of those with a less severe CML disease stage. Thus, take caution when interpreting aggregate results. Color-coded ✸ indicates significant Bonferroni-corrected (*p* < 0.00016) pairwise differences between two TKIs. (**A**). Aggregate percentage of patients with CCyR per TKI. (**B**). Aggregate percentage of patients with MMR per TKI. (**C**). Aggregate percentage of overall survival per TKI.

Figure 4 illustrates the analytical results examining the percentage of patients in CCyR (Figure 4A), percentage of patients in MMR (Figure 4B), and percentage of patients overall survived (%OS) for the subset studies that included functional metric(s). Pairwise statistical comparison identified significant differences in CCyR and MMR between TKI therapies (*p* < 0.0016) as denoted by the color-coded ✸ in Figure 4. OS over 12+ months was >90% for bosutinib, dasatinib, imatinib, and nilotinib, respectively, as shown in Figure 4C. In fact, the only pairwise significant difference in OS was with imatinib, which had a significantly higher OS. However, caution must be taken when interpreting aggregate % CCyR, %MMR, and %OS among the different TKIs due to inherent differences in the patients who were prescribed a specific TKI. Notably, many of the second-generation TKI studies had patients with more severe diseases, such as accelerated phase, blast crisis, resistant mutations, or had previously failed to appropriately respond to one or more TKIs, especially previous failure on imatinib. Thus, while imatinib does have significantly greater %CCyR and %OS, those patients treated successfully with imatinib tended to have less severe disease.

**Figure 5:**
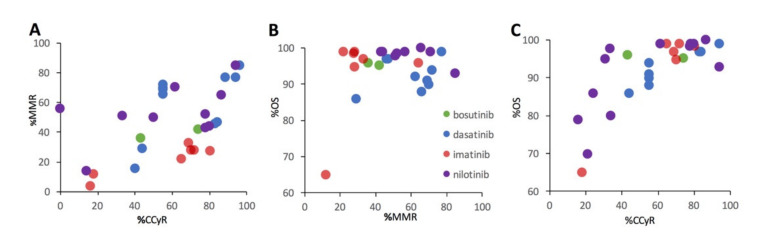
Unsupervised machine learning was used to assess clusters in a subset of CML TKI studies that included multiple functional metrics. Each data point represents a single study. Clustering revealed that percent of percent of patients with a major molecular response (%MMR) was not a good predictor of percent of patients overall survived (%OS) of 12+ months. %OS is more closely tied to the CML disease stage or the presence of a resistant mutation. (**A**). Percentage of patients with MMR (%MMR) versus percentage of patients with CCyR (%CCyR). (**B**). Percentage of patients overall survived (%OS) versus percentage of patients with MMR (%MMR). (**C**). Percentage of patients overall survived (%OS) versus percentage of patients with CCyR (%CCyR).

Unsupervised clustering was utilized to assess trends among functional metrics across heterogeneous CML populations, including %CCyR, %MMR, and %OS. The covariance in CCyR, MMR, and OS did not significantly depend on TKI therapy type. Rather, in the present dataset, CCyR and OS are more closely tied to the CML disease stage at treatment initiation (chronic phase versus accelerated phase or blast crisis) or the presence of a resistant mutation, than the specific type of TKI drug therapy. Figure 5 illustrates the relationships between %MMR and %CCyR (Figure 5A), %OS and % MMR (Figure 5B), and %OS and %CCyR for the subset of studies that included 2 or more functional metrics. There is a positive relationship between %MMR and %CCyR (Figure 5A). While there is a strong positive relationship between %OS and %CCyR (Figure 5C), the relationship between %OS and %MMR is much less apparent (Figure 5B).

## 4. Discussion

TKI adverse events were not the primary concern with the advent of “miraculous” TKIs like imatinib (Gleevec), dasatinib (Sprycel), nilotinib (Tasigna), and bosutinib (Bosulif) [10]. All TKIs have relatively high success in functionally treating CML when taken as prescribed. However, adherence is the key to long-term success, including preventing drug resistance and relapse. Thus, the present meta-analysis of GI AEs with CML TKI therapy provides important insights for improving optimal, personalized TKI selection to increase patient quality of life and TKI therapy adherence. Assessment of one of the largest heterogeneous CML aggregate populations to date in the present meta-analysis reveals some interesting differences in adverse events profiles compared to standard, more homogeneous drug approval studies alone, as discussed in detail below.

Imatinib was approved by the United States Food and Drug Administration (FDA) for adult Ph + CML patients in 2001 as an alternative to interferon α-2a (IFN-α). The clinical study used to support the approval of imatinib included 551 newly diagnosed patients taking 400 mg of imatinib once a day or twice a day [27]. In the initial imatinib approval study, 45% experienced diarrhea, 50% experienced nausea, and 23% experienced vomiting [27]. In the present meta-analysis, imatinib was only included as a comparative treatment arm within studies that were primarily analyzing a second-generation TKI. In fact, many of the included patients were switched due to imatinib intolerance or resistance. Nonetheless, even within the differing population constraints, the original imatinib trials had slightly higher GI AE incidence rates than the present meta-analysis where 24% of imatinib patients experienced diarrhea, 33% experienced nausea, and 16% experienced vomiting.

Nilotinib was approved by the FDA in 2018 [28]. The most commonly reported drug-related adverse reactions included nausea, diarrhea, and vomiting. Of the 279 newly diagnosed patients taking 300 mg of nilotinib twice a day, 14% of patients experienced diarrhea, 19% of patients experienced nausea, and 9% of patients of vomiting [28]. These incidence rates are higher than the rates of AEs in the present meta-analysis, which included 7%, 7%, and 13% for diarrhea, vomiting, and nausea, respectively.

Dasatinib was approved by the FDA in 2006 [29]. The clinical trial included a treatment arm of dasatinib and one of imatinib. Of 258 newly diagnosed patients taking a median average daily dose of 99 mg of dasatinib, 18% of patients experienced diarrhea, 9% experienced nausea, and 5% experience vomiting [29]. In the present meta-analysis, 28% of dasatinib patients experienced diarrhea, 19% experienced nausea, and 12% experienced vomiting.

Bosutinib was approved by the FDA in 2017 [30]. The bosutinib clinical trial showed high incidence rates of gastrointestinal adverse events, similar to the rates calculated in the present meta-analysis. For all grades out of 268 newly diagnosed CML patients taking 400 mg of bosutinib, 70% experienced diarrhea, 35% experienced nausea, and 18% experienced vomiting [30]. From the meta-analysis for bosutinib patients, 79% experienced diarrhea, 42% experienced nausea, and 37% experienced vomiting. Notably, the sample size for bosutinib (*n* = 881 patients) was the lowest of all the drugs included in this meta-analysis. Nonetheless, the sample size does not explain or nullify the large statistical differences in GI AEs when compared to the other TKIs.

The discrepancies of incidence rates between the approval studies and the meta-analysis could be, in part, dependent on dosage and habituation. Patients from the meta-analysis took similar doses of imatinib (400–800 mg/day) and nilotinib 300–400 mg/day compared to the approval studies [27,28]. However, for dasatinib, patients included in the meta-analysis took between 100–140 mg/day, whereas patients in the approval study had a median dose of 99 mg/day [29]. Smilarly, for bosutinib, patients in the meta-analysis had a dose of 500 mg/day whereas patients in the approval study had a dose of 400 mg/day [30]. The higher doses included in the present meta-analysis compared to the approval studies for dasatinib [29] and bosutinib [30] could be attributed to the higher GI AE rates in the meta-analysis. Additionally, all patients in the TKI FDA approval studies were newly diagnosed; however, the same is not the case for the meta-analysis. Patients included in the meta-analysis were on their first-, second-, or even third-line treatment, yielding a more heterogeneous patient population. These patients with a longer disease duration, who were not as naïve to treatment, could be more accustomed or habituated to the drug, possibly resulting in fewer GI AEs.

**Figure 6:**
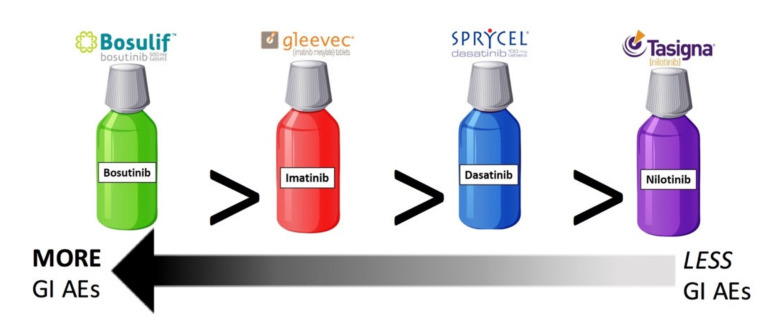
Summary depiction of meta-analysis results assessing gastrointestinal adverse events for the four tyrosine kinase inhibitors. There were significant differences in GI AE incidence among TKI types. Bosutinib had the most GI AEs and nilotinib the fewest GI AEs. Imatinib and dasatinib are the most comparable, although imatinib still has significantly more GI AEs than dasatinib.

The conducted heterogeneous meta-analysis had a sample size of 10,789 patients across 43 studies analyzed. As compared to the average sample size of 276 CML patients per peer-reviewed study, the sample size for the present meta-analysis is a factor of 40 larger. The present meta-analysis also included a heterogeneous patient population, unlike clinical trial populations, which are often homogenous, favoring younger and healthier patients [13]. Meta-analysis results show there are significant differences in GI AEs as a function of TKI type with the descending order of incidence of GI AEs per drug being bosutinib, imatinib, dasatinib, and nilotinib (Figure 6). Bosutinib definitively had the largest quantity and severity of GI AEs while nilotinib had the fewest GI AEs. Imatinib does have significantly more GI AEs than dasatinib, but these two TKIs were nonetheless the most comparable in their GI AE profiles.

A limitation of this meta-analysis was the availability of data on antecedent conditions and comorbidities for the included patients. Previously, guidelines for cancer treatment did not consider the disease interactions between cancer and comorbidity, and clinicians adopted a “single-disease” approach for treatment selection [31]. Data on treatment outcomes for patients with known comorbidities is lacking because patients with comorbidities are often excluded from clinical trials, yielding results that may not fully translate to the general cancer population. In the United States, about 40% of cancer patients report having at least one comorbidity or antecedent chronic condition [18]. As previously noted, the third most common comorbidity associated with CML is gastrointestinal disorders [18]. For this reason, GI AEs are an important consideration for TKI selection. For example, clinicians prescribing bosutinib to CML patients should be aware of GI comorbidities that could be exacerbated by the higher incidence rates of GI AEs associated with bosutinib.

TKI-related adverse events disrupt the lives of patients, which can reduce patient TKI adherence. Prior work suggests TKI non-adherence, defined as taking <80% of doses, could be impacting at least 30–40% of patients on prescribed TKI therapy [31,32]. Non-adherence could induce CML relapse for patients who are currently in remission [11,12,15,31,32,33]. Given that the relapse rate after prescribed, purposeful TKI cessation is between 50–60% [2,7], it can be hypothesized that lack of TKI compliance in patients with ongoing TKI treatment could yield similar results. Previous evidence supports a relationship between quality of life and adherence to therapy; TKI-related symptoms can decrease a patient’s quality of life, and in turn, potentially decrease compliance to treatment [31,32,33].

It should also be noted that gastrointestinal adverse events (GI AEs) are not the only adverse events identified with TKIs. While GI AEs are frequently cited by patients due to their often chronic or persistent nature, some acute adverse events that can be potentially life-threatening include changes in cardiovascular and pulmonary function [34,35]. Much like GI AEs, the incidence of other adverse events tends to vary as a function of specific TKI. For example, dasatinib has been more associated with pleural effusion and nilotinib with cardiac arrhythmias, such as prolonging the QT interval [34,35].

## 5. Conclusions

Gastrointestinal adverse events (GI AEs) due to TKI therapy may contribute to CML patient quality of life and decrease long-term TKI patient compliance. In this systematic review of 43 studies and 10,789 patients, bosutinib had the highest mean incidence of GI AEs, while nilotinib had the lowest incidence of GI AEs. Imatinib and dasatinib were the most comparable in their qualitative GI AE profile, although dasatinib had significantly less nausea and vomiting than imatinib. Assessment of functional metrics like complete cytogenetic response (CCyR), major molecular response (MMR), and overall survival (OS) suggested that while there are small significant differences between therapies, said differences are more attributed to study patient selection (i.e., CML disease stage, progression, or presence of resistant mutations) than the specific TKI drug utilized. Considering patients with more severe initial CML disease or with previously unresponsive CML progression comprised the majority of the patient populations for the stronger second-generation TKIs (bosutinib, dasatinib, nilotinib), there is likely no clinically meaningful difference in efficacy in the present meta-analysis. While this meta-analysis focused on gastrointestinal adverse events (GI AEs), TKIs are known to have other acute cardiovascular, pulmonary, and musculoskeletal adverse events as well. In conclusion, for patients with chronic phase CML without known TKI resistance, ongoing TKI therapy should be selected to minimize chronic and acute adverse events as a function of the TKI drug adverse event profile and the patient’s comorbidities and lifestyle.

## Figures and Tables

**Table 1:** Tabulated data for tyrosine kinase inhibitor (TKI) gastrointestinal adverse events (GI AEs) for the meta-analysis of 43 published studies and 10,789 chronic myeloid leukemia (CML) patients.

Adverse Event	Bosutinib	Dasatinib	Imatinib	Nilotinib
Diarrhea (all)	698/881 (79.2%)	792/2815 (28.1%)	282/1181 (23.9%)	313/4398 (7.1%)
Diarrhea (severe)	84/881 (9.5%)	76/2696 (2.8%)	20/1352 (1.5%)	14/2775 (0.5%)
Vomiting (all)	328/881 (37.2%)	273/2249 (12.1%)	167/1049 (15.9%)	272/3860 (7.0%)
Vomiting (severe)	27/881 (3.1%)	16/2249 (0.7%)	4/1220 (0.3%)	15/1958 (0.8%)
Nausea (all)	372/881 (42.2%)	514/2680 (19.2%)	346/1049 (33.0%)	514/3881 (13.2%)
Nausea (severe)	10/881 (1.1%)	21/2680 (0.8%)	3/1220 (0.2%)	19/2327 (0.8%)

## Data Availability

All data is publicly available in the original included studies, which are listed in Supplementary Table S1. All aggregate data used for statistical analysis is provided in Table 1 and the figures of the article.

## Supplementary Materials

The following are available online at https://www.mdpi.com/article/10.3390/cancers13071643/s1, Figure S1: Text mining to identify adverse events from literature text, Figure S2: PRISMA diagram illustrating meta-analysis study inclusion and exclusion criteria, Table S1: Table of included studies in the meta-analysis.
