# Supplementary material for: Meta-Analysis of Gastrointestinal Adverse Events from Tyrosine Kinase Inhibitors for Chronic Myeloid Leukemia
Source: Cancers (Basel). 2021 Apr 1;13(7):1643. doi: 10.3390/cancers13071643 (PMC8037766; doi:10.3390/cancers13071643)
Supplement: Supplementary file 1 [file cancers-13-01643-s001.pdf]

cancers

Supplementary Materials: Meta-Analysis of Gastrointestinal Adverse Events from Tyrosine Kinase Inhibitors for Chronic Myeloid Leukemia

Prahathishree Mohanavelu, Mira Mutnick, Nidhi Mehra, Brandon White, Sparsh Kudrimoti, Kaci Hernandez Kluesner, Xinyu Chen, Tim Nguyen, Elaina Horlander, Helena Thenot, Vamsi Kota and Cassie S. Mitchell

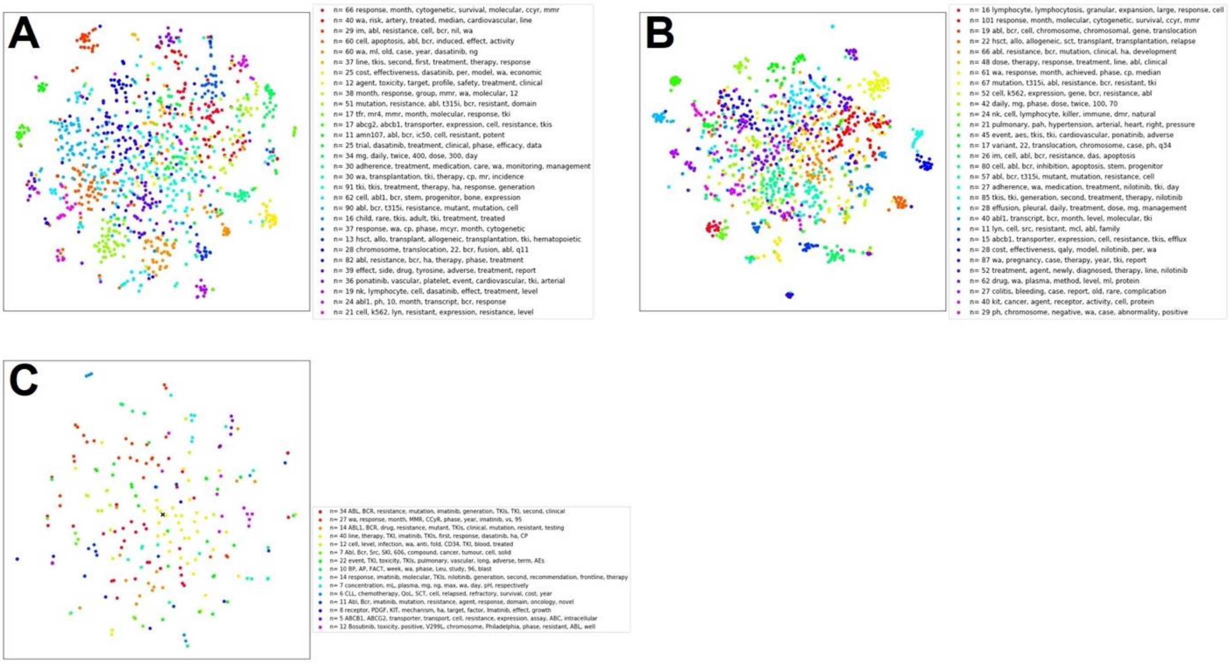

Figure S1. Text mining to identify adverse events from literature text. Text mining was performed to assess top breadth and sufficient sample size. Broad field wide plots mapping commonly occurring term cluster within abstracts containing "chronic myeloid leukemia" and a specific treatment ("dasatinib", "nilotinib", or "bosutinib") were created to use in topic decision. Abstracts were lemmatized and weighted with term frequency-inverse document frequency scores to eliminate commonly occurring, non-informative words. The k-means clustering algorithm was applied to the TF-IDF matrices, and clusters containing articles which had similar groups of significant terms were formed. Dimensionality of the clusters was reduced using singular value decomposition followed by t-stochastic neighbor embedding (t-SNE) to allow for visualization. Panel A shows the clustering for abstracts containing "chronic myeloid leukemia" and "dasatinib" (n = 1325 abstracts). Panel B shows the clustering for abstracts containing "chronic myeloid leukemia" and "bosutinib" (n = 232 abstracts). Panel C shows the clustering for abstracts containing "chronic myeloid leukemia" and "nilotinib" (n = 1166 abstracts). A notable cluster is a cluster (n = 27 abstracts) with 'colitis' and 'bleeding' in Panel A.

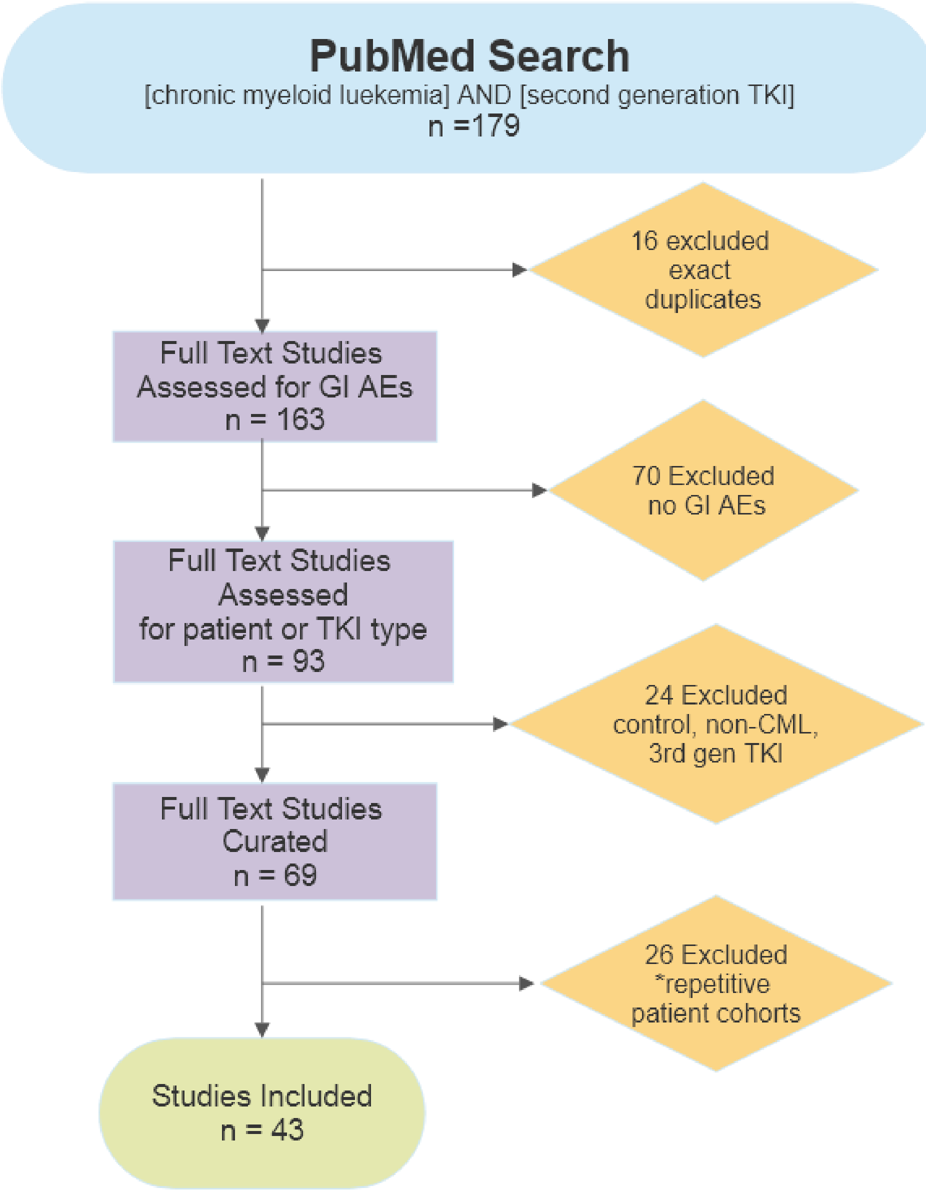

Figure S2. PRISMA diagram illustrating meta-analysis study inclusion and exclusion criteria. Details are give in the Methods section of the main manuscript. Note that "repetitive" studies include patient cohorts that are secondarily analyzed by follow-up publications. Only the original or most complete unique patient cohort meeting inclusion criteria is included in the final set of studies for analysis.

Table S1. Table of included studies in the meta-analysis. Includes all data sources where data was extracted and analyzed. Full citation reference corresponding to the numbered reference for each study under the "Citation" column is given below the table.

PMID	Author	Total Number of Patients	Age	Type of Age (Mean/Median)	Citation
16775234	Talpaz et al.	84	56	Median	[1]
17138817	Hocchaus et al.	186	59	Median	[2]
17185463	Cortes et al.	116	52–55	Median	[3]
17264298	Guilhot et al.	107	57	Median	[4]
17317857	Kantarjian et al.	150	51	Median	[5]
17715389	Kantarjian et al.	280	58	Median	[6]
18048643	Coutre et al.	119	57	Median	[7]
18754032	Cortes et al.	157	49.5–55	Median	[8]
19263190	Sakamaki et al.	54	43–64	Median	[9]
19280591	Quintas-Cardama et al.	138	57	Median	[10]
19369231	Kantarjian et al.	317	56	Median	[11]
19449194	Tojo et al.	34	62	Median	[12]
19536906	Kantarjian et al.	150	51	Median	[13]
19729517	Garg et al.	48	52	Median	[14]
19822896	Rosti et al.	73	51	Median	[15]
20139391	Shah et al.	670	54–56	Median	[16]
20520639	Giles et al.	60	58–62	Median	[17]
20525993	Saglio et al.	846	46–47	Median	[18]
20525995	Kantarjian et al.	519	46–49	Median	[19]
21732337	Nicolini et al.	1422	53	Median	[20]
22076466	Coutre et al.	137	57	Median	[21]
22271898	Pemmaraju et al.	846			[22]
22915637	Radich et al.	246	47–50	Median	[23]
24333114	O'Dwyer et al.	60	54	Median	[24]
24345751	Kantarjian et al.	570	53	Median	[25]
25196702	Brummendorf et al.	502	47–48	Median	[26]
25519749	Yeung et al.	210	48.5	Median	[27]
25540064	Nakaseko et al.	63	55	Median	[28]
25703064	Murai et al.	54	63.5	Median	[29]
25766724	Wang et al.	267	39–41	Median	[30]
26437782	Hocchaus et al.	1089	53	Median	[31]
26993758	Cortes et al.	52	51.7	Mean	[32]
27509035	Takahashi et al.	174	47–49	Median	[33]
27771544	Miyamura et al.	45	47	Median	[34]
28218239	Hocchaus et al.	190	55	Median	[35]
28550414	Shiseki et al.	16	50	Mean	[36]
28699641	Hughes et al.	421	48	Median	[37]
28795321	Hara et al.	31	55.2	Mean	[38]
28895203	Murai et al.	79	62	Median	[39]
29058817	Kumagai et al.	54	56	Median	[40]
29362980	Ishikawa et al.	38	57.5	Median	[41]
29556695	Kim et al.	39	57	Median	[42]
29713954	Noguchi et al.	76	54.5–55	Median	[43]

References

1. Talpaz, M., et al., Dasatinib in imatinib-resistant Philadelphia chromosome-positive leukemias. *N. Engl. J. Med.* **2006**, 354, p. 2531–2541.

2. Hochhaus, A., et al., Dasatinib induces notable hematologic and cytogenetic responses in chronic-phase chronic myeloid leukemia after failure of imatinib therapy. *Blood* **2007**, *109*, p. 2303–2309.
3. Cortes, J., et al., Dasatinib induces complete hematologic and cytogenetic responses in patients with imatinib-resistant or -intolerant chronic myeloid leukemia in blast crisis. *Blood* **2007**, *109*, p. 3207–3213.
4. Guilhot, F., et al., Dasatinib induces significant hematologic and cytogenetic responses in patients with imatinib-resistant or -intolerant chronic myeloid leukemia in accelerated phase. *Blood* **2007**, *109*, p. 4143–4150.
5. Kantarjian, H., et al., Dasatinib or high-dose imatinib for chronic-phase chronic myeloid leukemia after failure of first-line imatinib: a randomized phase 2 trial. *Blood* **2007**, *109*, p. 5143–5150.
6. Kantarjian, H.M., et al., Nilotinib (formerly AMN107), a highly selective BCR-ABL tyrosine kinase inhibitor, is effective in patients with Philadelphia chromosome-positive chronic myelogenous leukemia in chronic phase following imatinib resistance and intolerance. *Blood* **2007**, *110*, p. 3540–3546.
7. le Coutre, P., et al., Nilotinib (formerly AMN107), a highly selective BCR-ABL tyrosine kinase inhibitor, is active in patients with imatinib-resistant or -intolerant accelerated-phase chronic myelogenous leukemia. *Blood* **2008**, *111*, p. 1834–1839.
8. Cortes, J., et al., Efficacy and safety of dasatinib in imatinib-resistant or -intolerant patients with chronic myeloid leukemia in blast phase. *Leukemia* **2008**, *22*, p. 2176–2183.
9. Sakamaki, H., et al., Phase 1/2 clinical study of dasatinib in Japanese patients with chronic myeloid leukemia or Philadelphia chromosome-positive acute lymphoblastic leukemia. *Int. J. Hematol.* **2009**, *89*, p. 332–341.
10. Quintas-Cardama, A., et al., Bleeding diathesis in patients with chronic myelogenous leukemia receiving dasatinib therapy. *Cancer* **2009**, *115*, p. 2482–2490.
11. Kantarjian, H., et al., Phase 3 study of dasatinib 140 mg once daily versus 70 mg twice daily in patients with chronic myeloid leukemia in accelerated phase resistant or intolerant to imatinib: 15-month median follow-up. *Blood* ***2009***, *113*, p. 6322–6329.
12. Tojo, A., et al., A Phase I/II study of nilotinib in Japanese patients with imatinib-resistant or - intolerant Ph+ CML or relapsed/refractory Ph+ ALL. *Int. J. Hematol.* **2009**, *89*, p. 679–688.
13. Kantarjian, H., et al., Dasatinib or high-dose imatinib for chronic-phase chronic myeloid leukemia resistant to imatinib at a dose of 400 to 600 milligrams daily: two-year follow-up of a randomized phase 2 study (START-R). Cancer, 2009. 115(18): p. 4136-47.
14. Garg, R.J., et al., The use of nilotinib or dasatinib after failure to 2 prior tyrosine kinase inhibitors: long-term follow-up. *Blood*. **2009**, *114*, p. 4361–4368.
15. Rosti, G., et al., Nilotinib for the frontline treatment of Ph(+) chronic myeloid leukemia. *Blood* **2009**, *114*, p. 4933–4938.
16. Shah, N.P., et al., Potent, transient inhibition of BCR-ABL with dasatinib 100 mg daily achieves rapid and durable cytogenetic responses and high transformation-free survival rates in chronic phase chronic myeloid leukemia patients with resistance, suboptimal response or intolerance to imatinib. *Haematologica* **2010**, *95*, p. 232-240.
17. Giles, F.J., et al., Nilotinib is active in chronic and accelerated phase chronic myeloid leukemia following failure of imatinib and dasatinib therapy. *Leukemia* **2010**, *24*, p. 1299–1301.
18. Saglio, G., et al., Nilotinib versus imatinib for newly diagnosed chronic myeloid leukemia. N Engl J Med, 2010. 362(24): p. 2251-9.
19. Kantarjian, H., et al., Dasatinib versus imatinib in newly diagnosed chronic-phase chronic myeloid leukemia. *N. Engl. J. Med.* **2010**, *362*, p. 2260–2270.
20. Nicolini, F.E., et al., Expanding Nilotinib Access in Clinical Trials (ENACT): an open-label, multicenter study of oral nilotinib in adult patients with imatinib-resistant or imatinib-intolerant Philadelphia chromosome-positive chronic myeloid leukemia in the chronic phase. *Cancer* **2012**, *118*, p. 118–126.
21. le Coutre, P.D., et al., Nilotinib in patients with Ph+ chronic myeloid leukemia in accelerated phase following imatinib resistance or intolerance: 24-month follow-up results. *Leukemia* **2012**, *26*, p. 1189–1194.
22. Pemmaraju, N., et al., Analysis of outcomes in adolescents and young adults with chronic myelogenous leukemia treated with upfront tyrosine kinase inhibitor therapy. *Haematologica* **2012**, *97*, p. 1029–1035.
23. Radich, J.P., et al., A randomized trial of dasatinib 100 mg versus imatinib 400 mg in newly diagnosed chronic-phase chronic myeloid leukemia. *Blood* **2012**, *120*, p. 3898–3905.
24. O'Dwyer, M.E., et al., Nilotinib 300 mg BID as frontline treatment of CML: prospective analysis of the Xpert BCR-ABL monitor system and significance of 3-month molecular response. *Leuk. Res.* **2014**, *38*, p. 310–315.
25. Kantarjian, H.M., et al., Bosutinib safety and management of toxicity in leukemia patients with resistance or intolerance to imatinib and other tyrosine kinase inhibitors. *Blood* **2014**, *123*, p. 1309–1318.
26. Brummendorf, T.H., et al., Bosutinib versus imatinib in newly diagnosed chronic-phase chronic myeloid leukaemia: results from the 24-month follow-up of the BELA trial. *Br. J. Haematol.* **2015**, *168*, p. 69–81.
27. Yeung, D.T., et al., TIDEL-II: first-line use of imatinib in CML with early switch to nilotinib for failure to achieve time-dependent molecular targets. *Blood* **2015**, *125*, p. 915–923.
28. Nakaseko, C., et al., A phase 1/2 study of bosutinib in Japanese adults with Philadelphia chromosome-positive chronic myeloid leukemia. *Int. J. Hematol.* **2015**, *101*, p. 154–164.
29. Murai, K., et al., A prospective analysis of clinical efficacy and safety in chronic myeloid leukemiachronic phase patients with imatinib resistance or intolerance as evaluated using European LeukemiaNet 2013 criteria. *Eur. J. Haematol.* **2015**, *95*, p. 558–565.
30. Wang, J., et al., Phase 3 study of nilotinib vs imatinib in Chinese patients with newly diagnosed chronic myeloid leukemia in chronic phase: ENESTchina. *Blood* **2015**, *125*, p. 2771–2778.
31. Hochhaus, A., et al., Frontline nilotinib in patients with chronic myeloid leukemia in chronic phase: results from the European ENEST1st study. *Leukemia* **2016**, *30*, p. 57–64.

32. Cortes, J.E., et al., Evaluating the Impact of a Switch to Nilotinib on Imatinib-Related Chronic Low-Grade Adverse Events in Patients With CML-CP: The ENRICH Study. *Clin. Lymphoma. Myeloma. Leuk.* **2016**, *16*, p. 286–296.
33. Takahashi, K., et al., A propensity score matching analysis of dasatinib and nilotinib as a frontline therapy for patients with chronic myeloid leukemia in chronic phase. *Cancer* **2016**, *122*, p. 3336–3343.
34. Miyamura, K., et al., Switching to nilotinib in patients with chronic myeloid leukemia in chronic phase with molecular suboptimal response to frontline imatinib: SENSOR final results and BIM polymorphism substudy. *Leuk. Res.* **2016**, *51*, p. 11–18.
35. Hochhaus, A., et al., Treatment-free remission following frontline nilotinib in patients with chronic myeloid leukemia in chronic phase: results from the ENESTfreedom study. *Leukemia* **2017**, *31*, p. 1525–1531.
36. Shiseki, M., et al., Dasatinib rapidly induces deep molecular response in chronic-phase chronic myeloid leukemia patients who achieved major molecular response with detectable levels of BCR-ABL1 transcripts by imatinib therapy. *Int. J. Clin. Oncol.* **2017**, *22*, p. 972–979.
37. Hughes, T.P., et al., Nilotinib dose-optimization in newly diagnosed chronic myeloid leukaemia in chronic phase: final results from ENESTxtnd. *Br. J. Haematol.* **2017**, *179*, p. 219–228.
38. Hara, R., et al., NKG2D gene polymorphisms are associated with disease control of chronic myeloid leukemia by dasatinib. *Int. J. Hematol.* **2017**. *106*, p. 666–674.
39. Murai, K., et al., Rapid reduction in BCR-ABL1 transcript predicts deep molecular response in dasatinib-treated chronic-phase chronic myeloid leukaemia patients. *Eur. J. Haematol.* **2018**, *100*, p. 27–35.
40. Kumagai, T., et al., Dasatinib cessation after deep molecular response exceeding 2 years and natural killer cell transition during dasatinib consolidation. *Cancer. Sci.* **2018**, *109*, p. 182–192.
41. Ishikawa, J., et al., Efficacy and safety of switching to nilotinib in patients with CML-CP in major molecular response to imatinib: results of a multicenter phase II trial (NILSw trial). *Int. J. Hematol.* **2018**, *107*, p. 535–540.
42. Kim, D.W., et al., Outcomes of switching to dasatinib after imatinib-related low-grade adverse events in patients with chronic myeloid leukemia in chronic phase: the DASPERSE study. *Ann. Hematol.* **2018**, *97*, p. 1357–1367.
43. Noguchi, S., et al., Switching to nilotinib is associated with deeper molecular responses in chronic myeloid leukemia chronic phase with major molecular responses to imatinib: STAT1 trial in Japan. *Int. J. Hematol*. **2018**, *108*, p. 176–183.
